# Metabolic Regulation by p53 Family Members

**DOI:** 10.1016/j.cmet.2013.06.019

**Published:** 2013-11-05

**Authors:** Celia R. Berkers, Oliver D.K. Maddocks, Eric C. Cheung, Inbal Mor, Karen H. Vousden

**Affiliations:** 1 The CR-UK Beatson Institute, Glasgow G61 1BD, Scotland, UK

## Abstract

The function of p53 is best understood in response to genotoxic stress, but increasing evidence suggests that p53 also plays a key role in the regulation of metabolic homeostasis. p53 and its family members directly influence various metabolic pathways, enabling cells to respond to metabolic stress. These functions are likely to be important for restraining the development of cancer but could also have a profound effect on the development of metabolic diseases, including diabetes. A better understanding of the metabolic functions of p53 family members may aid in the identification of therapeutic targets and reveal novel uses for p53-modulating drugs.

## Main Text

**Figure 1:**
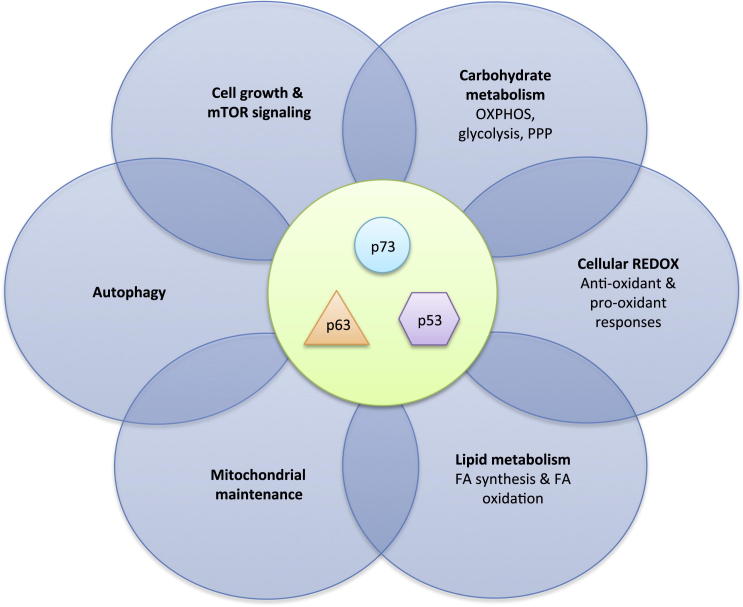
Outline of the Interaction between p53 Family Members and Metabolic Pathways p53 and its family members p63 and p73 have been implicated in many aspects of cellular metabolism, including AMPK and mTOR signalling, carbohydrate and lipid metabolism, the regulation of autophagy, and the maintenance of mitochondrial integrity and REDOX balance.

The transcription factor p53 is best known for its role as a tumor suppressor, and a wealth of evidence underscores the importance of p53 in inhibiting cancer development (Vousden and Prives, 2009). Mice lacking p53 are prone to the development of early-onset spontaneous tumors (Donehower et al., 1992). In most human cancers, p53 function is lost (Hollstein et al., 1991), whereas patients that inherit one mutant *TP53* allele display an enormously increased cancer risk, a condition known as Li-Fraumeni syndrome (Varley, 2003). As a key component in the cellular response to stress, p53 is activated by numerous intrinsic and extrinsic stress signals, including genotoxic damage, oncogene activation, loss of normal cell contacts, and nutrient or oxygen deprivation—many of which may be encountered during malignant transformation (Horn and Vousden, 2007). The outcome of the p53-mediated stress response depends on cell type and context as well as the extent, duration, and origin of the stress. Severe or sustained stress accompanied by irreversible damage, such as extreme genotoxic damage or the activation of oncogenes, results in the induction of cell death or senescence. Such responses effectively eliminate the affected cells, thus limiting the inappropriate accumulation of cells with heritable genomic damage and inhibiting malignant development. On the other hand, mild stress results in a subtler p53 response consistent with repairing or preventing damage. In such cases, p53 may engage antioxidant responses in order to decrease ROS levels or participate in DNA damage repair processes while inducing a transient cell-cycle arrest, thereby allowing cells to survive safely until the damage has been resolved. Transient metabolic stresses—such as fluctuations in oxygen or nutrient availability—also trigger a more adaptive response, in which p53 induces metabolic remodelling and promotes catabolism, while coordinating a decrease in proliferation and cell growth (Figure 1) (Jones et al., 2005; Scherz-Shouval et al., 2010; Maddocks et al., 2013). These metabolic functions of p53 are emerging as important components of the p53 response that not only aid in maintaining normal cellular homeostasis but also contribute to the control of tumor development. The mechanisms through which p53 is activated by metabolic and other stress signals are complex and have been reviewed elsewhere (Kruse and Gu, 2009). Once activated, p53 primarily exerts its functions by acting as a transcription factor, regulating the expression of both genes and microRNAs (miRNAs). p53 has also been reported to possess cytosolic functions. For example, cytoplasmic p53 has been reported to inhibit autophagy (Tasdemir et al., 2008; Morselli et al., 2009; Maiuri et al., 2010), interact with Bcl2 family members to promote apoptosis (reviewed in Green and Kroemer, 2009), promote necrosis by opening the mitochondrial permeability transition pore (Vaseva et al., 2012), and regulate glucose metabolism by binding to glucose-6-phosphate dehydrogenase (G6PDH) (Jiang et al., 2011). Hence, the outcome of p53 activation is complicated, and cell-fate decisions are mediated by transcription-dependent and -independent responses that can vary according to the level and posttranslational modifications of p53 and its interaction with other proteins, including other transcription factors (we will return to this theme later).

Alterations in metabolism are increasingly regarded as essential for tumor progression, and many reports suggest that tumor cells become dependent on this metabolic remodelling for their growth and survival (Vander Heiden et al., 2009; Ward and Thompson, 2012). Given its central role as a tumor suppressor, it is not surprising that p53 can regulate several aspects of cellular metabolism and thereby counteract many of the metabolic alterations associated with cancer development (Figure 1). p53 interacts with mammalian target of rapamycin (mTOR) and AMP-activated protein kinase (AMPK), two master regulators of cellular metabolism, directly influences many key pathways involved in carbohydrate and lipid metabolism, and regulates autophagy and the oxidative stress response. Recently, metabolic roles have also been ascribed to the p53 family members p63 and p73, further broadening the impact of the p53 family on cell metabolism. In this review, we discuss how p53 and its family members regulate cellular metabolism through mechanisms that are not only crucial for restraining the development of cancer but could also profoundly influence other aspects of health and disease, including aging and the development of metabolic disease.

### Control of Metabolic Pathways by the p53 Family

#### The Interplay between p53 and the Cell’s Metabolic Sensors

**Figure 2:**
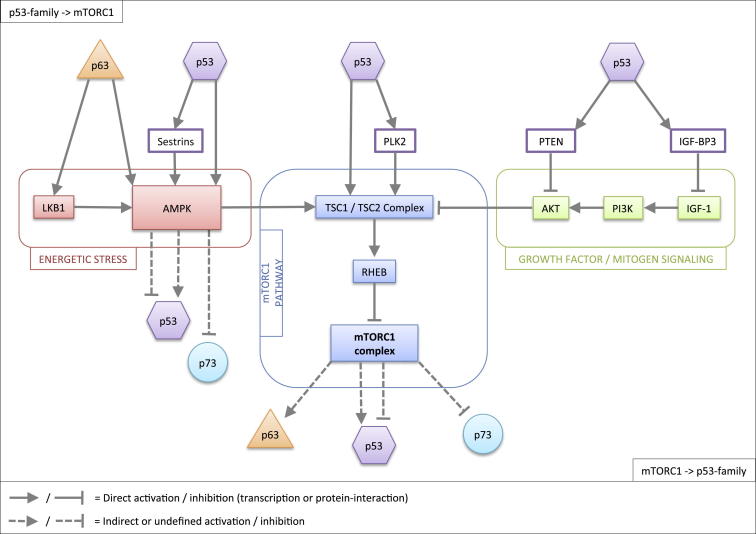
Outline of the Crosstalk between p53 Family Members and the mTORC1 Pathway p53 and p63 act upstream of mTORC1 and inhibit mTORC1 activity via multiple transcriptional targets. p53, p63, and p73 can all be modulated by the AMPK/mTORC1 pathway (indicated in red and blue, respectively) and link mTORC1 signalling to multiple downstream effects, including, but not limited to, cell-cycle control (via p53), autophagy (via p73), and differentiation (via p63).

The mTOR protein is an important positive regulator of cell growth and proliferation that can influence the development of diabetes, aging, and cancer (Zoncu et al., 2011). mTOR forms two multimeric protein complexes, each with distinct functions (Laplante and Sabatini, 2009). The mTORC1 complex (consisting of mTOR, Deptor, mLST8 [GβL], PRAS40, and Raptor) has been studied extensively. Its downstream effectors control cell growth and energy metabolism by regulating protein translation and synthesis, mitochondrial biogenesis, lipid synthesis, and autophagy (Howell and Manning, 2011). The functions of mTORC2 (consisting of mTOR, Deptor, mLST8 [GβL], Rictor, Sin1, and Protor-1) are less well defined but include the regulation of the cytoskeleton (Jacinto et al., 2004) and cell survival (Berchtold and Walther, 2009). mTORC1 is active in the presence of both adequate growth conditions (the availability of nutrients, oxygen, and energy) and mitogens that positively signal for cell division. Conversely, mTORC1 is inhibited by the absence of nutrients or adequate growth conditions and by cellular stresses, which can introduce genomic damage during the process of cell division (Sengupta et al., 2010). Consequently, a number of important signaling pathways converge on and coregulate mTORC1 activity (Figure 2), including the IGF/AKT/PI3K growth-factor-signaling pathway and the p53 stress-signaling pathway. The presence of nutrients and energy can be sensed by mTORC1 via various mechanisms. For example, energetic stress signals to mTORC1 via the cellular fuel sensor AMPK. The activation of AMPK (in response to an increase in the AMP to ATP ratio) exerts an inhibitory effect on mTORC1 (Hardie et al., 2012) and coordinates activities that allow cells to adapt to metabolic stress. mTORC1 can also be activated in an amino-acid-dependent manner. The presence of amino acids stimulates the recruitment of mTORC1 to the late endosomal and lysosomal compartments, which enables mTORC1 to interact with sensors of growth factor signaling (Zoncu et al., 2011). By integrating amino acid sensing with growth factor signaling, this mechanism ensures that mTORC1 is only activated in the presence of both.

Given the influence of mTORC1 signaling, it is unsurprising that there are multiple points of crosstalk with the p53 pathway, providing a reciprocal network that is integral to cellular homeostasis (Figure 2). p53 is activated in response to stress, so it generally exerts an inhibitory effect upstream on mTORC1 in order to shut down cell growth, cell division, and energy consumption under adverse conditions. p53 promotes the expression of sestrins, which can activate AMPK (Budanov and Karin, 2008), and thereby inhibit mTORC1 (Feng et al., 2005). Other transcriptional targets of p53 that can negatively regulate mTORC1 include *AMPKβ*, *TSC2*, *IGF-BP3*, *PTEN*, and *Plk2*, all of which are induced by genotoxic stress (Feng et al., 2007; Matthew et al., 2009). Nongenotoxic p53 activation by nutlin-3a has also been shown to lead to mTOR inhibition via AMPK activation (Drakos et al., 2009). Although nongenotoxic activation of p53 initially induces a cell-cycle arrest, the ability of p53 to inhibit mTOR in parallel is important in determining the eventual outcome of the p53 response. Cells that sustain mTOR activity (such as through the deletion of *TSC2*) progress to irreversible senescence, a process named geroconversion, whereas cells in which mTOR is inhibited (for example, through p53 activation or under hypoxia) ultimately achieve a reversible quiescent state (Korotchkina et al., 2010; Leontieva et al., 2012). The intriguing suggestion here is that, whereas p53 promotes quiescence, it suppresses geroconversion and senescence (by inhibiting mTOR), which may contribute to tumor suppression by preventing the induction of senescence-associated cancer-promoting responses (Blagosklonny, 2012). The fact that AMPK can act both upstream and downstream of p53 adds another level of complexity to the p53-mTORC1-AMPK signaling pathways. As described above, during genotoxic stress and nongenotoxic activation of p53, AMPK can be activated downstream of p53 to inhibit mTORC1. However, during energetic stress, AMPK acts upstream of p53, and the inhibition of mTORC1 (by AMPK) occurs in concert with the AMPK-dependent activation of p53 via serine-15 phosphorylation (Imamura et al., 2001; Jones et al., 2005). This signal establishes a p53-dependent G_1_-S checkpoint that prevents S phase entry when cellular energy supplies are inadequate to support the considerable demands of cell division, resulting in a transient cell-cycle arrest. Furthermore, in hepatocellular carcinoma cells, AMPK has been shown to exert an inhibitory effect on the p53 deacetylase Sirt1, thereby enhancing p53 acetylation and activation (Lee et al., 2012a). These studies suggest that AMPK can activate p53 via various mechanisms. However, AMPK has also been shown to activate Sirt1 and enhance the deacetylation of Sirt1 targets in skeletal muscle cells (Cantó et al., 2009), indicating that the ability of AMPK to activate p53 via this route may be tissue- and context-dependent.

Cellular homeostasis necessitates reciprocal and flexible signaling between the mTOR and p53 pathways in order to balance adequate stress response with the requirement for growth and proliferation (Feng and Levine, 2010). Indeed, like AMPK, mTORC1 not only acts downstream of p53 but can also influence p53 activity. For example, survival signaling through the Notch1 receptor, constitutive active forms of which have oncogenic activity, activates mTORC1 via PI3K and AKT, resulting in the inhibition of p53 activity by eIF4E, a translation initiation factor downstream of mTORC1 (Mungamuri et al., 2006). Conversely, constitutive mTORC1 activation has been shown to activate p53 by enhancing p53 translation (Lee et al., 2007) and inducing the expression of alternative reading frame (ARF), a small protein that inhibits MDM2, thus stabilizing p53 in response to oncogene activation (Miceli et al., 2012). This suggests that abnormal signaling through mTOR (a sign of malignant transformation) activates p53 (Feng and Levine, 2010). Interestingly, AKT-induced senescence occurs via mTORC1-dependent regulation of p53 translation and stabilization of p53 protein (Astle et al., 2012), further supporting the notion that oncogenic signaling can activate p53 via mTOR. When a more restrained p53 response is required (for example, in response to energetic stress), a feedback mechanism involving both AMPK and mTOR may aid in mounting a transient and self-limiting p53 response. p53 activation by 5-Aminoimidazole-4-carboxamide ribonucleotide (AICAR), an activator of AMPK, has been shown to be attenuated by the inhibition of mTORC1, suggesting that a self-limiting feedback loop exists whereby AMPK activation simultaneously triggers rapid p53 activation (via serine-15 phosphorylation) and inhibition of mTOR, which eventually shuts down p53 translation (Lee et al., 2007). However, such experiments should be interpreted with caution, given that AICAR is an intermediate in nucleotide synthesis, the imbalance of which has been shown to activate p53 (Linke et al., 1996). During some forms of metabolic stress, the mTOR and p53 pathways may also operate independently (Maddocks et al., 2013). Therefore, it is vital to view and interpret the interplay between p53 and mTOR with careful consideration for the specific context and tissue type.

#### The Regulation of Central Carbon Metabolism by p53

Glucose is a major carbon source for mammalian cells. Once it is taken up by the cell, glucose is broken down to pyruvate in the cytosol, a process known as glycolysis, which yields a limited amount of ATP. In most normal (quiescent) cells, pyruvate is subsequently fed into the mitochondrial tricarboxylic acid (TCA) cycle in order to generate NADH and FADH_2_, which, in turn, can be used for further ATP production via the highly efficient process of oxidative phosphorylation (OXPHOS). However, the majority of cancer cells display alterations in glucose metabolism (Ward and Thompson, 2012). Often, glycolysis is the preferred pathway for producing energy, even under normal aerobic conditions, and pyruvate is converted primarily to lactate. This shift from mitochondrial respiration to aerobic glycolysis is known as the Warburg effect, and this effect is thought to help satisfy the altered metabolic needs of tumor cells. Their high rate of proliferation requires not only increased amounts of energy but also the rapid production of building blocks necessary for growth and the control of oxidative stress for maximal survival during growth (Beaconsfield and Reading, 1964; Boros et al., 1998). Intermediates from glycolysis can serve as precursors for de novo amino acid, lipid, and nucleotide synthesis and to generate reducing agents that are crucial for maintaining the cellular redox state. For example, an important secondary pathway that branches from glycolysis is the pentose phosphate pathway (PPP), which uses glucose-6-phosphate in reactions that ultimately produce both NADPH and ribose-5-phosphate (R5P). NADPH is an essential reducing agent in many biosynthetic reactions, including de novo lipid synthesis, and helps to protect against oxidative stress by regenerating reduced glutathione (GSH). R5P is a critical component in the de novo synthesis of nucleotides and nucleic acids. The role of the PPP is particularly evident in cells or tissues that undergo proliferation, such as cancer tissues (Beaconsfield et al., 1965; Wamelink et al., 2008; for review see Tong et al., 2009) but also, for example, in regenerating liver tissues (Beaconsfield et al., 1965; Ledda-Columbano et al., 1985). Furthermore, the importance of the PPP in cancer is highlighted in various drug resistance studies that showed that drug-resistant cancer cells often have an elevated PPP activity in comparison to drug-sensitive cells (Gessner et al., 1990; Fanciulli et al., 1993; Ferretti et al., 1993; McBrayer et al., 2012); therefore, the inhibition of the PPP can often inhibit tumor growth (Boros et al., 1997; Ramos-Montoya et al., 2006; McBrayer et al., 2012). Increased expression of some PPP enzymes, such as transketolase, has been suggested to be an indicator for poorer cancer patient survival (Langbein et al., 2006; McBrayer et al., 2012). Further down the glycolytic pathway, glyceraldehyde-3-phosphate is an essential building block for the production of triacylglycerols and phospholipids, whereas 3-phosphoglycerate (3-PG) can be diverted to the serine synthesis pathway in order to contribute to the production of nucleotides as well as various nonessential amino acids, which can also serve as substrates for GSH and phospholipid synthesis.

**Figure 3:**
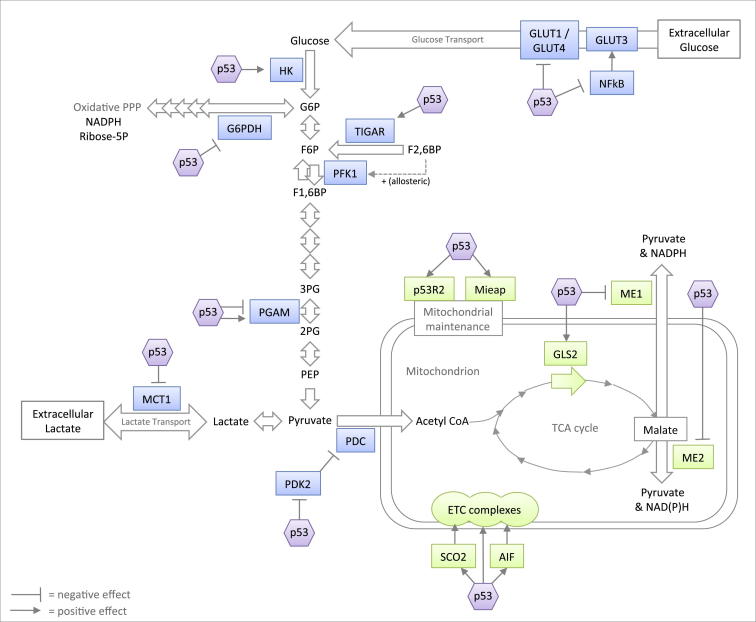
Outline of the Regulation of Central Carbohydrate Metabolism by p53 p53 generally dampens aerobic glycolysis (blue) and promotes mitochondrial respiration (green) through multiple mechanisms, although it can both positively and negatively modulate PPP activity.

p53 regulates both glycolysis and oxidative phosphorylation and also modulates PPP activity (Figure 3). True to its role as a tumor suppressor, p53 has been described to counteract the Warburg effect by dampening aerobic glycolysis and promoting oxidative phosphorylation through multiple mechanisms. Phosphofructose kinase 1 (PFK1), which catalyzes the third step of the glycolytic pathway, is allosterically regulated by various metabolites in the glucose metabolism pathways. Metabolites such as ATP, citrate, and lactate, which indicate an adequate supply of energy, directly inhibit PFK1, whereas PFK1 can be activated by AMP and fructose-2,6-bisphosphate (F2,6BP). p53 plays a critical role in this pathway by inducing the expression of *TP53*-induced glycolysis and apoptosis regulator (TIGAR), which acts as a phosphatase that degrades F2,6BP and thereby decreases the activity of PFK1. Hence, p53, via TIGAR, lowers the glycolytic rate and would be predicted to promote the diversion of glycolytic intermediates into the PPP (Li and Jogl, 2009; Bensaad et al., 2006). Other glycolytic enzymes are also inhibited by p53 (Figure 3). In fibroblasts, protein levels of phosphoglycerate mutase (PGAM), which catalyzes the conversion of 3-PG to 2-PG, are downregulated by p53 (Kondoh et al., 2005). p53 also negatively regulates the expression of pyruvate dehydrogenase kinase 2 (PDK2), which inactivates the pyruvate dehydrogenase complex (PDC), a protein that converts pyruvate to acetyl-CoA. Hence, p53 activates PDC, thereby favoring the production of acetyl-CoA at the expense of lactate production (Contractor and Harris, 2012). In addition to regulating glycolytic enzymes, p53 decreases intracellular glucose levels by inhibiting glucose uptake. The expression of the glucose transporters GLUT1 and GLUT4 is directly downregulated by p53 (Schwartzenberg-Bar-Yoseph et al., 2004), whereas p53 can also modulate the NF-κB pathway in order to regulate glycolytic flux (Kawauchi et al., 2008b). Although p53 enhances the DNA-binding activity of NF-κB, it also suppresses its transcriptional activity by inhibiting the activity of IKKβ (Kawauchi et al., 2008a, 2009). The net result of these opposing p53 activities appears to be the inhibition of NF-κB activity, which results in decreased GLUT3 expression (Kawauchi et al., 2008b). Furthermore, the repression of monocarboxylate transporter 1 (MCT1) expression by p53 prevents the efflux of lactate under hypoxic conditions, which also dampens glycolytic rates (Boidot et al., 2012). However, some of the described activities of p53 seem to enhance rather than inhibit glycolysis. For example, the promoters of both PGAM and hexokinase II (HKII, which catalyzes the first step of glycolysis) contain p53-responsive elements (Mathupala et al., 1997; Ruiz-Lozano et al., 1999). The exact mechanisms underlying these apparently opposing p53 activities are largely unknown. To some extent, the regulation of the glycolytic pathway by p53 is likely to be tissue- and context-dependent (Vousden and Ryan, 2009; Maddocks and Vousden, 2011). Indeed, a muscle-specific isoform of PGAM is transcriptionally activated by p53 in cardiocytes (Ruiz-Lozano et al., 1999), which is in contrast to the p53-dependent destabilization of PGAM protein in fibroblasts (Kondoh et al., 2005). Opposing p53 responses have also been reported in the p53-dependent regulation of the PPP. The simultaneous activation of hexokinase II and inhibition of PFK-1 (via the expression of TIGAR) would lead to an increased flux into the PPP. In contrast, p53 was recently described to inhibit the diversion of glycolytic intermediates into the PPP by binding and inhibiting G6PDH, the enzyme that catalyzes the first and rate-limiting step of the PPP. These seemingly contradictory roles of p53 in regulating the PPP most likely reflect, at least in part, the differential p53 response to different types of stress. Oxidative stress necessitates the upregulation of PPP activity in order to increase NADPH production and support the antioxidant response, and TIGAR has indeed been shown to be critical in protection against oxidative stress (Bensaad et al., 2006), metabolic stress (Bensaad et al., 2009; Wanka et al., 2012), and hypoxia (Cheung et al., 2012). TIGAR-deficient mice are more sensitive to acute intestinal injuries, and the growth defects of TIGAR-null cells can be rescued by ROS scavengers and nucleotides (Cheung et al., 2013). On the other hand, by downregulating PPP activity in developing cancer cells, p53 may counteract the production of the building blocks that are critical for growth and proliferation and thereby hinder tumor development.

The restriction of glycolytic flux by p53 is paralleled by the ability of p53 to drive OXPHOS and help maintain mitochondrial integrity (Figure 3). p53 has been described to regulate mitochondrial DNA copy number and mitochondrial mass (Lebedeva et al., 2009; Kulawiec et al., 2009) via the induction of p53R2 expression (p53-controlled ribonucleotide reductase) (Kulawiec et al., 2009; Bourdon et al., 2007). p53 regulates mitochondrial quality control by inducing the repair or removal of damaged mitochondria (mitophagy) through the induction of mitochondria-eating protein (Mieap) (Kitamura et al., 2011). Furthermore, synthesis of cytochrome c oxidase 2 (SCO2) —a key component involved in OXPHOS required for the assembly of the cytochrome c oxidase (COX) complex (complex IV in the mitochondrial electron transport chain (ETC) and the major site of oxygen consumption in the mitochondria)—is transcriptionally activated by p53 (Matoba et al., 2006), as is subunit I of the COX complex (Okamura et al., 1999). A third target that is transcriptionally activated by p53 in this context is apoptosis-inducing factor (AIF) (Stambolsky et al., 2006), which is essential for the maintenance of mitochondrial complex I (Vahsen et al., 2004). In addition to promoting OXPHOS, other activities of p53 may increase the TCA cycle rate. For example, p53 transcriptionally activates glutaminase 2 (GLS2) (Hu et al., 2010; Suzuki et al., 2010), a mitochondrial glutaminase that catalyzes the hydrolysis of glutamine to glutamate, which can fuel the TCA cycle after its conversion to α-ketoglutarate. p53 also transcriptionally represses the expression of malic enzymes ME1 and ME2, which recycle malate to pyruvate, and p53 could thereby inhibit the utilization of TCA cycle intermediates into biosynthetic pathways and NADPH production (Jiang et al., 2013). In contrast to its role in maintaining mitochondrial health under mild stress or nonstressed conditions, p53 represses PGC-1α and PGC-1β, transcriptional cofactors that are master regulators of mitochondrial biogenesis, under conditions of extreme stress, such as telomere shortening. This leads to mitochondrial dysfunction, which—along with induction of the classical p53 functions (senescence, apoptosis, and cell-cycle arrest)—has been proposed to contribute to aging (Sahin et al., 2011).

#### p53 as a Regulator of Lipid Metabolism

**Figure 4:**
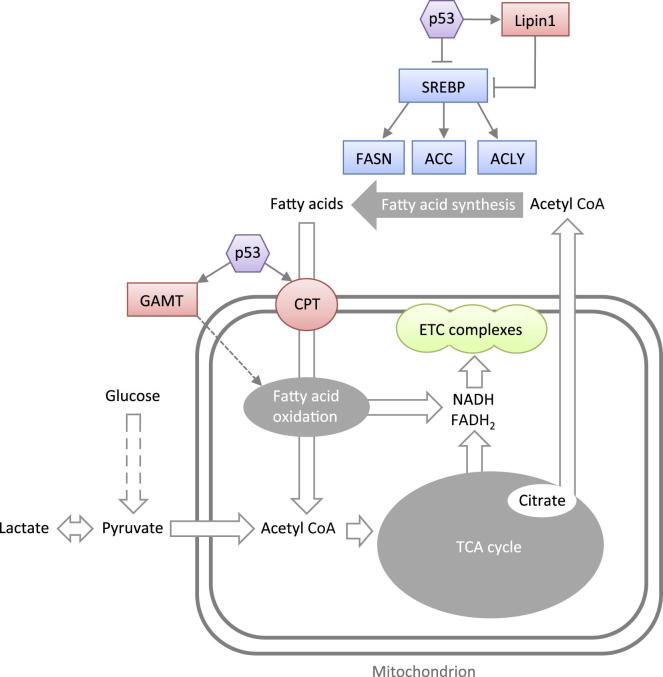
Outline of the Regulation of Lipid Metabolism by p53 p53 generally functions as a negative regulator of lipid synthesis by enhancing fatty acid oxidation (red) and inhibiting fatty acid synthesis (blue) through multiple mechanisms.

Lipid metabolism is a highly regulated and synchronized process. Fatty acids can be used by cells as an energy source or a means of storing surplus energy. Fatty acid oxidation (FAO) takes place in the mitochondria and breaks down fatty acids into two-carbon units in order to yield acetyl-CoA, NADH, and FADH_2_, which can be channelled directly to the TCA cycle and electron transport chain to produce ATP (Figure 4). Fatty acid synthesis takes place in the cytosol and uses two-carbon units to form a gradually elongating carbon chain in a process that requires ATP and NADPH. Thus, to avoid futile cycling, these reciprocal pathways are separated into different cellular compartments and regulatory mechanisms at various levels ensure that they do not occur simultaneously. De novo fatty acid synthesis occurs mainly in a limited number of tissues—i.e., adipose tissue, the liver, and lactating mammary glands. Fatty acids are transported to cells throughout the body through the circulation in the form of lipoprotein particles, which are made up of newly synthesized fatty acids as well as dietary fatty acids, and are exported from adipose, liver, or gut tissues. When supply is higher than demand, fatty acids can be stored in liver and fat tissue in lipid droplets, mainly in the form of triglycerides. Another important branch of lipid metabolism is the mevalonate pathway, through which the two-carbon acetyl groups are utilized to synthesize cholesterol. Whereas adult differentiated cells obtain lipids mainly from the diet, highly proliferative cells such as embryonic stem cells and cancer cells display high rates of de novo fatty acid synthesis for generating building blocks for new membranes as the cells divide and for steroid hormones that enhance cell growth (Swinnen et al., 2006; Santos and Schulze, 2012). Multiple tumor types display elevated levels of cholesterol or lipid droplets. Expression of key enzymes in the fatty acid synthesis pathway, such as fatty acid synthase (FASN), acetyl CoA carboxylase (ACC), and ATP citrate lyase (ACLY), was shown to be reactivated in various tumors and contribute to cell transformation and tumorigenesis (Swinnen et al., 2006). Additionally, the PI3K-AKT and mTOR pathways, which are commonly activated in cancer cells (Yang et al., 2002; Santos and Schulze, 2012), are known to enhance lipid synthesis. Finally, under hypoxic conditions that are expected to occur in solid tumors, FAO is inhibited because NADH and FADH_2_ cannot be oxidized and, thus, such conditions shift the balance toward lipid synthesis and accumulation (Santos and Schulze, 2012).

p53 plays a crucial role in lipid metabolism, participating in both normal and pathological conditions. True to its role as a tumor suppressor, p53 generally functions as a negative regulator of lipid synthesis by activating fatty acid oxidation and inhibiting fatty acid synthesis (Figure 4). Mouse embryonic fibroblasts that can be induced to differentiate into adipocytes provide a well-described model for the study of lipogenic cells. In this model system, the role of p53 as a negative regulator of adipogenesis is apparent—activated p53 inhibits adipocyte differentiation (Hallenborg et al., 2009), and p53 knockout enhances lipid accumulation (Molchadsky et al., 2008; Wang et al., 2013). The mechanisms by which p53 exerts an antilipogenic effect on cells seem complex and are probably multifaceted. Lipid metabolism is controlled by the mTOR pathway (Figure 2) and is tightly linked to glycolysis and the PPP, which supply the building blocks (acetyl-CoA and NADPH) needed for lipid synthesis (Figure 3). Thus, modulation of these pathways by p53 contributes to an altered lipogenic status of the cell. p53 has also been demonstrated to directly affect the expression of proteins involved in lipid metabolism (Figure 4) (an extensive review of lipid-metabolism-related genes regulated by p53 can be found in Goldstein and Rotter, 2012). For example, p53 induces the expression of carnitine acetyltransferases (such as CPT1C), proteins that are responsible for the transport of fatty acids into the mitochondria for FAO (Zaugg et al., 2011). p53 has also been demonstrated to regulate key transcription factors responsible for the expression of genes involved in determining cellular lipogenic status, such as sterol regulatory element-binding protein 1 (SREBP1)—a key transcription factor for genes involved in fatty acid synthesis. p53 suppresses the expression of the SREBP1c isoform in mouse adipose tissue, contributing to the repression of FASN and ACLY (Yahagi et al., 2003). After glucose starvation, p53 induces guanidinoacetate methyltransferase (GAMT), an enzyme involved in creatine synthesis, which facilitates increased FAO through mechanisms that are still unknown (Ide et al., 2009). Under similar conditions, p53 activation by ROS induces lipin1 (a negative regulation of SREBP activity) in C2C12 myoblasts, which also results in enhanced FAO, whereas knockdown of p53 attenuates the increase in FAO in response to glucose starvation (Assaily et al., 2011). Inhibition of mTORC1 has been shown to promote the nuclear entry of lipin1, a process that could further contribute to the p53-mediated repression of SREBP activity (Peterson et al., 2011). Although the consequences of p53-mediated promotion of FAO may allow cells to adapt and survive when glucose is no longer available, the ATP generated in response to GAMT activation by p53 has also been shown to support apoptosis (Ide et al., 2009).

#### p53 and the Regulation of ROS

The regulation by p53 of carbohydrate and lipid metabolism is tightly linked to another important function of p53: the regulation of ROS. In its role as a “guardian of the cell,” p53 can eliminate the deleterious effects of oxidative insult either by limiting ROS damage in cells that can be salvaged or by using ROS to eliminate cells damaged beyond repair. Hence, if the insult is transient and repairable, then p53 can activate a suite of antioxidant responses, many of which are linked to carbohydrate or lipid metabolism. For example, the activation of the PPP via TIGAR produces NADPH that can reduce glutathione, an important cellular antioxidant. Interestingly, a recent study showed that p53 can also directly promote GSH synthesis at the expense of nucleotide synthesis after serine deprivation, and, thereby, it actively controls ROS levels under conditions of metabolic stress (Maddocks et al., 2013). Mitochondria are a major source of ROS, especially when they are damaged. The downregulation of AIF, for example, leads to enhanced levels of ROS that are due to defective mitochondrial function (Klein et al., 2002). Hence, by maintaining mitochondrial integrity, basal p53 activity may also limit ROS production. In addition, p53 transcriptionally activates antioxidant genes to offer protection against damage. Examples of p53-induced anti-ROS proteins include members of the sestrin family (Budanov and Karin, 2008), aldehyde dehydrogenase (ALDH4) (Yoon et al., 2004), and TP53INP1 (Cano et al., 2009). p53 can also act as an inhibitor to repress the expression of pro-oxidant genes such as nitric oxide synthase (NOS2) (Ambs et al., 1998) and cyclooxygenase 2 (COX2) (Subbaramaiah et al., 1999). On the other hand, if the damage is irreparable, p53 may evoke a pro-oxidant state, which will ensure the demise of the damaged cell (see Zhuang et al., 2012, for an extensive review of pro-oxidant genes activated by p53). For example, p53 has been shown to activate genes such as those encoding proline oxidase (PIG6/POX) and ferredoxin reductase (Liu and Chen, 2002; Rivera and Maxwell, 2005) and to inhibit proteins (directly or by transcriptional repression) involved in the antioxidant response, such as G6PDH, malic enzymes, and manganese superoxide dismutase (Zhao et al., 2005; Jiang et al., 2011; Jiang et al., 2013). Although the general effect of pro-oxidant functions of p53 is related to enhanced cell death, the consequences of p53-mediated control of ROS are complicated by the requirement of ROS for proliferative as well as cell-death signaling. In this context, the ability of p53 to activate the Nox2 complex for the production of ROS has been associated with redox-sensitive signaling that mediates proliferation and survival (Italiano et al., 2012). How different stresses result in different p53-dependent ROS responses is not exactly known, but the interaction of p53 with other proteins and transcription factors is most likely one of the factors that mediates this process. Posttranslational modifications on p53 (such as phosphorylation or acetylation) or simply the amount of p53 (low basal levels of p53 or higher amounts of activated p53) may influence the nature of these interactions. For example, basal p53 levels have been described to increase catalase activity and decrease ROS under physiological conditions, whereas p53 that is activated by genotoxic stress switches to inhibit catalase activity, leading to a pro-oxidant state (Kang et al., 2013). Also, the interaction between p53 and Nrf2 (a master regulator of the oxidative stress response), which we will discuss in more detail later, is likely to be important in determining the outcome of the p53-dependent regulation of ROS.

#### The Roles of p53 in Regulating Autophagy

Autophagy (here referring to macroautophagy), which literally translates as “self-eating,” is an important cellular catabolic process carried out in the cytoplasm. Autophagy is characterized by the formation of double-membraned autophagosomes around cytoplasmic components that have been targeted for degradation. Once enclosed around their cargo, autophagosomes fuse with lysosomes, forming autolysosomes in which the delivered contents are then catabolized (Rubinsztein et al., 2012). Autophagy fulfils two basic functions important to normal cellular homeostasis: the removal of aged or dysfunctional organelles and proteins and the release of nutrients liberated from the degraded macromolecules. Autophagy is upregulated in response to cellular stresses, including nutrient deprivation (Mordier et al., 2000) and oxidative stress (Kiffin et al., 2004). Although it was initially thought that autophagy was responsible for a form of cell death, this hypothesis has been revised in light of a large body of evidence supporting autophagy as a prosurvival mechanism (Levine and Kroemer, 2009). Autophagy is involved in a wide range of pathologies, including metabolic, neurodegenerative, and inflammatory diseases as well as cancer (Rubinsztein et al., 2012). It has been proposed that, although basal levels of autophagy have a tumor suppressor function, stress-responsive autophagy could promote the survival of tumor cells faced with metabolic stress (Morselli et al., 2009; Scherz-Shouval et al., 2010).

The relationship between p53 and autophagy is complex, given that p53 can both promote and inhibit autophagy in a context-dependent manner (Maiuri et al., 2010; Morselli et al., 2009; Maddocks and Vousden, 2011). This ability to suppress or enhance autophagy may allow p53 to provide the most appropriate cell-survival strategy during nutrient starvation (Scherz-Shouval et al., 2010); in mouse embryonic fibroblasts with low basal autophagy, the effect of p53 is to increase autophagy during starvation. Conversely, in HCT116 cells, in which the basal autophagic flux is high, p53 inhibits autophagy by downregulating LC3 mRNA at the posttranscriptional level in response to starvation in order to promote limited but sustainable levels of autophagy over time (Scherz-Shouval et al., 2010). It has also been proposed that the intracellular location of p53 activity dictates these functions. Nuclear (activated) p53 most likely activates autophagy (via transcriptional activation of autophagy-inducing proteins), whereas basal levels of cytoplasmic p53 directly inhibit autophagy (Maiuri et al., 2010; Morselli et al., 2009; Tasdemir et al., 2008).

The activation of autophagy by (nuclear) p53 is well established and can occur through a variety of mechanisms. The mTORC1 pathway is a major inhibitory regulator of autophagy. Hence, by negatively regulating mTORC1 (Figure 2), p53 activates autophagy. Furthermore, several p53 targets activate autophagy independently of mTOR. *DRAM-1* is activated by p53 and encodes multiple isoforms that regulate autophagy (Crighton et al., 2006; Mah et al., 2012). The proapoptotic p53 target PUMA has been shown to induce selective mitochondrial autophagy (mitophagy), which is dependent on Bax (Yee et al., 2009). Recently, ISG20L1 has been identified as a transcriptional target of all three p53 family members that upregulates autophagy in response to genotoxic stress (Eby et al., 2010). The tumor suppressor protein ARF has been shown to activate autophagy by p53-dependent and -independent means (Abida and Gu, 2008; Reef and Kimchi, 2008). Recently, the p53-inducable gene *Ei24* was found to be an essential component of the basal autophagy pathway in neurons and hepatocytes and to regulate autophagy under nonstressed conditions (Zhao et al., 2012), suggesting that p53 may play a homeostatic role in promoting autophagy. Interestingly, a reciprocal regulation of p53 by at least one ATG protein (autophagy-related gene, a family of proteins that play critical roles in the autophagy process) has recently been described. In addition to its role in the lipidation of LC3 (ATG8) (Tanida et al., 2012), ATG7 promotes p53-dependent transcription of p21^CDKN1A^ (encoding p21, an inhibitor of cyclin-dependent kinases), thereby promoting cell-cycle arrest and survival in response to nutrient starvation (Lee et al., 2012b).

The role of cytoplasmic p53 in the regulation of autophagy was established by suppressing p53 expression in colon cancer (HCT116) cells. Surprisingly, this resulted in the upregulation of autophagy, whereas the reintroduction of p53 repressed autophagy (Tasdemir et al., 2008). In similar experiments, the expression of cancer-relevant p53 mutants in p53^−/−^ HCT116 cells also inhibited autophagy, the mutants showing the highest cytoplasmic localization having the greatest effect (Morselli et al., 2008). It seems that the ability of p53 to suppress autophagy depends (at least in part) on complex formation with components and regulators of the autophagic machinery in either the cytoplasm or the nucleus. One example of this is HMGB1, a chromatin-binding factor with nuclear and cytoplasmic functions that interacts with p53 (Livesey et al., 2012). Under normal conditions, HMGB1 and p53 are localized to the nucleus in complexes that prevent the release of either protein to the cytoplasm. Loss of p53 causes the translocation of HMGB1 to the cytosol, where it interacts with ATG6 (Beclin1) to promote autophagy. The reintroduction of p53 suppresses this process and, therefore, inhibits autophagy (Livesey et al., 2012).

#### p53’s Interaction with Other Transcription Factors

Clearly, the identification of an ever-increasing number of functions for p53 provokes the important question of how the final response of the cell is determined. Many of the examples discussed above indicate that the particular outcome of the activation of p53 depends on the level of the insult. This may influence the activity of p53 itself as well as that of other proteins or transcriptional factors that ultimately determine the fate of the cell. p53 has clear relationships with other transcription factors that are known to be critical in the response to metabolic stress, most notably PGC, SREBP, and Nrf2. PGC-1α is a critical regulator of glucose, lipid, and energy metabolism, whose functions include the regulation of mitochondrial biogenesis and the expression of antioxidants. Under conditions of mild metabolic stress, p53 has been shown to increase PGC-1α expression, resulting in the induction of an antioxidant response (Aquilano et al., 2013). Moreover, a recent study demonstrated that PGC-1α modulates the transcriptional activity of p53 under conditions of glucose starvation to boost its metabolic function. This results in the recruitment of p53 to proarrest and metabolic genes, including p21 and TIGAR, leading to antioxidant responses, cell-cycle arrest, and survival. On the other hand, prolonged starvation resulted in the degradation of PGC-1α, leading to a proapoptotic p53 response (Sen et al., 2011). p53 also interacts with SREBPs, critical regulators of the genes that control lipid and sterol synthesis. Although wild-type (WT) p53 can repress SREBP1c-dependent gene expression in mouse adipose tissue (Figure 4) (Yahagi et al., 2003), tumor-associated mutant p53 has been shown to bind and transcriptionally activate SREBP2 in breast cancer cells, resulting in the induction of the mevalonate pathway (Freed-Pastor et al., 2012). Although the effect of SREBPs on p53-dependent gene expression has not yet been explored, it is possible that p53 and SREBP will mutually regulate each other. Finally, recent studies describe a complicated connection between p53 and Nrf2. Pharmacological activation of Nrf2 has been shown to suppress tumor progression. However, constitutively activated Nrf2 also contributes to drug resistance and tumor cell survival (Sporn and Liby, 2012), an effect that may be mediated by protection against ROS and an increase in anabolic pathways, including the PPP, purine synthesis pathways, and glutaminolysis (Mitsuishi et al., 2012). p53 has been shown to both enhance and repress Nrf2 activity; low basal p53 levels enhance Nrf2 protein levels to promote survival, and high stress levels result in the inhibition of Nrf2 by p53 and the induction of ROS and apoptosis (Chen et al., 2012). Similarly, p53 levels have been shown to be both positively and negatively regulated by Nrf2, and it has been suggested that Nrf2 and p53 synergize in enhancing the antioxidant response (Rotblat et al., 2012). The mechanisms by which p53 can both enhance and repress Nrf2 are still largely unknown. The enhancement of Nrf2 protein levels by p53 is dependent on p21 (a transcriptional target of p53) (Chen et al., 2012), which has been reported to stabilize and activate Nrf2 by binding to KEAP1, thereby inhibiting the proteasomal degradation of Nrf2 (Chen et al., 2009). It has also been shown that p53 can directly interact with certain promoter elements of Nrf2 target genes, resulting in the transcriptional repression of these genes (Faraonio et al., 2006), but additional studies are needed in order to fully elucidate the underlying mechanisms of these seemingly opposing actions of p53. Although the complicated interactions between p53 and metabolic transcription factors such as PGC, SREBP, and Nrf2 are still poorly understood, they may prove to be key to understanding the different outcomes to p53 activation in response to different types of stress. It is also tempting to speculate that the interaction with these transcription factors may help ensure that the extent and desired outcomes of the p53 response are coordinated with the extent of metabolic remodelling induced by p53. This may ensure channeling of the available cellular ATP stores to where they are needed most during different conditions. For example, if the energy-demanding process of DNA damage repair is desirable, then the required energy may be generated via the simultaneous p53-dependent remodelling of energy-generating pathways. Another example may be the simultaneous activation of both cell death and autophagy by p53 during severe stress. Given that autophagy is essentially a prosurvival mechanism, it remains puzzling why two seemingly opposed processes should be concomitantly active. A possible alternative explanation may be that autophagy accompanies p53-induced apoptosis to ensure an adequate supply of metabolic precursors (i.e., for energy production), without which this active, energy-dependent process could stall.

#### The p53 Family: p63 and p73 in Metabolism

p53 belongs to a family of transcription factors that also includes p63 and p73, functional homologs of p53 that show high sequential and structural similarity. Both the *Tp63* and *Tp73* genes are transcribed from two distinct promoters, resulting in either full-length proteins that retain a full transactivation (TA) domain (TAp63 and TAp73) or N-terminally truncated isoforms (ΔNp63 and ΔNp73) that lack part of this domain but still retain some ability to activate transcription. In addition, alternative RNA splicing results in different C termini for both p63 and p73 isoforms, termed α-ε and α-η, respectively. TAp63 and TAp73 can supplement p53 function by transactivating p53 target genes, resulting in cell-cycle arrest and apoptotic cell death, although p63 and p73 also have distinct transcriptional targets. In contrast, ΔNp63 and ΔNp73 generally have antiapoptotic functions and have been shown to act as dominant negatives for inhibiting the function of p53 family members. However, ΔN isoforms also activate specific sets of target genes and function independently of other p53 family isoforms. Despite these similarities and overlap in activity, p63 and p73 each have functions that are strikingly different from those of p53, and both proteins play critical roles in development (reviewed in Allocati et al., 2012). For example, *Tp63*^−*/*−^ mice show defects in the development of epithelial tissues as well as truncated limbs, and p63 also appears to be essential for the maintenance of stem cells. In humans, mutations in p63 cause ectodermal dysplasias, syndromes characterized by defects of ectodermal structures (such as hair and teeth). Unlike p63, p73 is essential for proper neural development, and p73 knockout mice display developmental defects in the CNS. Analysis of selective TAp73 or ΔNp73 knockdown suggested that ΔNp73 isoforms are necessary for neuronal survival, whereas TAp73 is required for the long-term maintenance and differentiation of neuronal stem cells. Both p63 and p73 are important for germ cell maintenance. p63 controls the quality of the female germline by eliminating damaged oocytes, whereas p73 maintains genomic stability of the oocyte pool.

A number of recent studies have shown that both p63 and p73 function in the regulation of different aspects of metabolism. Like p53, p63 interacts with both AMPK and the mTOR pathway (Figure 2), and the deregulation of p63 has been shown to affect lipid metabolism (Sabbisetti et al., 2009; Su et al., 2012). Activation of mTORC1 induces TAp63 and ΔNp63 expression, which form part of an mTOR/p63/Notch signaling cascade that can influence cell differentiation (Ma et al., 2010). TAp63 positively regulates the transcription of *Sirt1*, *AMPKα2*, and *LKB1* and thereby coordinates fat and glucose metabolism. Loss of TAp63 results in defects in lipid utilization, fatty acid synthesis, and FAO, and mice lacking *TAp63* consequently display insulin resistance as well as symptoms of obesity, type 2 diabetes, and premature aging (Su et al., 2012). ΔNp63α has been shown to transcriptionally activate FASN in both transformed and immortalized epithelial cells, and the maintenance of fatty acid synthesis contributes to the prosurvival activity of p63 during development (Sabbisetti et al., 2009). In addition, isoforms of p63 may play a role in mediating the metabolic response to drug treatment. For example, TAp63γ levels are elevated in response to treatment with metformin (a drug widely used to treat type 2 diabetes that reduces insulin resistance via mechanisms that include the inhibition of mitochondrial respiratory chain complex I, which ultimately results in the activation of AMPKα), and TAp63γ was crucial for the metformin-induced activation of AMPKα (Su et al., 2012). Recent work with squamous cell carcinoma cells suggests a significant role for ΔNp63α as a multifaceted regulator of stress-induced autophagy. Cisplatin treatment leads to phosphorylation of ΔNp63α by ataxia telangiectasia mutated (ATM; a protein involved in the response to DNA damage that can also activate p53), which resulted in phospho-ΔNp63α dependent expression of several genes in the *ATG* family (Huang et al., 2012b). Furthermore, multiple phospho-ΔNp63α responsive miRNAs were found to modulate the activity of ATG proteins, suggesting that ΔNp63-induced autophagy could contribute to cisplatin resistance. Interestingly, ΔNp63α and phospho-ΔNp63α induced different sets of metabolic target genes upon cisplatin treatment, including genes involved in carbohydrate and lipid metabolism (Huang et al., 2012a).

Like p53, p73 is a major transcriptional regulator of autophagy (Crighton et al., 2007); but, whereas p53 influences autophagy by acting upstream of mTORC1, p73 expression is negatively regulated downstream of mTORC1 (Figure 2) (Rosenbluth et al., 2008). Hence, p73 is activated when mTOR signaling is inhibited and targets multiple autophagosome- and lysosome-associated genes to promote autophagy (Rosenbluth et al., 2008; Rosenbluth and Pietenpol, 2009). Given that the activation of autophagy is a major feature of mTOR inhibition, this places p73 as an important player in the response to cellular stress and makes it possible that signals initiated by p53 eventually direct p73 activity. Another difference between p53 and p73 in the regulation of autophagy is the role of DRAM-1. Although p73 (like p53) positively regulates DRAM-1, p73-induced autophagy is independent of DRAM-1 activation and is not associated with cell death (Crighton et al., 2007). Interestingly, ΔNp73 can inhibit p53- and p73-dependent stress-induced autophagy, but not starvation-induced autophagy (Crighton et al., 2007), suggesting the presence of multiple autophagic pathways that are differentially regulated by p73. The inhibition of p73 by mTORC1 also controls several genes involved in insulin response (Rosenbluth et al., 2008; Rosenbluth and Pietenpol, 2009) as well as genes and miRNAs involved in mesenchymal differentiation and tumorigenesis (Rosenbluth et al., 2011). In addition, p73 is negatively regulated by AMPK via direct interaction with AMPKα, which selectively represses the p73 transcriptional program (Lee et al., 2009). Finally, TAp73 has been implicated in the regulation of mitochondrial respiration. Cytochrome c oxidase subunit 4 is a TAp73 target, and the depletion of TAp73 resulted in a decrease in the activity of mitochondrial complex IV, paralleled with decreased oxygen consumption and ATP production and increased levels of ROS. Consequently, TAp73^−/−^ mice showed signs of premature aging associated with increased oxidative damage and senescence (Rufini et al., 2012).

### Physiological Relevance of the Metabolic Functions of the P53 Family

#### Cancer

The canonical functions of p53—the induction of cell-cycle arrest, senescence, and apoptotic cell death—have long been regarded as the key mechanisms by which p53 inhibits tumor development, but this view is being increasingly challenged. Mice lacking PUMA, a p53 target gene that is critical in mediating p53’s apoptotic activity in many tissues (Yu and Zhang, 2008), fail to develop early-onset spontaneous tumors (Michalak et al., 2008). Similarly, p53^25,26^, a p53 mutant that retains the ability to induce senescence, but not p53-mediated cell-cycle arrest and apoptosis, still inhibited KrasG12D-induced lung carcinogenesis (Brady et al., 2011). Although the p53 target gene *p21*^*CDKN1A*^ is critical for inducing p53-dependent senescence and cell-cycle arrest, mice lacking p21 are not prone to develop early-onset tumors (Choudhury et al., 2007). Even mice that express p53^3KR^, a mutant that has lost p53-dependent cell-cycle arrest, apoptosis, and senescence, are protected from early-onset tumorigenesis (Li et al., 2012). Intriguingly, p53^3KR^ retains some of the metabolic functions of WT p53; i.e., it can regulate metabolic target genes such as GLS2, GLUT3, and TIGAR, resulting in decreased ROS levels and the suppression of glucose uptake and glycolytic flux. This suggests that the metabolic functions of p53 may be central to p53’s role as a tumor suppressor, especially when its canonical functions are compromised. A recent study revealing that the regulation of TCA cycle enzymes by p53 strongly induced senescence further underscored the interdependence of the canonical and metabolic functions of p53 in tumor suppression (Jiang et al., 2013).

So, how do the metabolic functions of p53 contribute to tumor suppression? Some help to prevent the accumulation of heritable genomic damage and, therefore, hinder tumor development. Examples of such functions are the coordination of cell growth and proliferation via mTOR and AMPK, the ability to lower ROS levels and restore the redox balance, and the activation of autophagy to remove aged or dysfunctional organelles (Figure 1). In addition, p53 directly opposes many aspects of the metabolic transformation that seem crucial for malignant transformation and cancer progression. For example, p53 directly counteracts the Warburg effect by dampening glycolysis and promoting oxidative phosphorylation. By inhibiting PPP activity, opposing fatty acid synthesis, and inducing FAO, p53 opposes anabolism and prevents cells from adopting a more lipogenic status (Figure 1). Hence, regulating the metabolic state of the cell may serve as an independent mechanism by which p53 can restrain tumor development. This multilayer protection afforded by p53 can be regarded as additional insurance for preventing tumor development, but some of p53’s prosurvival roles are not always easily reconciled with tumor suppression. The ability of p53 to lower ROS levels, for example, might help established tumor cells to survive oxidative stress. Similarly, the role of p53 in limiting glycolysis also promotes the diversion of glycolytic intermediates into anabolic pathways such as the PPP. Also, p53-induced activation of autophagy could be beneficial for tumor growth under conditions of metabolic stress. Normally, these prosurvival p53 activities would be tightly controlled in order to avoid the activation of an improper response, but, clearly, hijacking the metabolic functions of p53 under conditions of sustained stress—in which repair or recovery is not possible—could help rather than hinder tumor development. Indeed, the presence of p53 has been shown to protect tumor cells from metabolic stress induced by glucose or serine starvation (Jones et al., 2005; Maddocks et al., 2013). The regulation of autophagy by p53 has been shown to promote tumor cell survival under conditions of chronic nutrient deprivation (Scherz-Shouval et al., 2010), and p53 protects cancer cells against treatment with metformin, an inhibitor of mitochondrial respiratory chain complex I (Buzzai et al., 2007), although a recent study did not show enhanced sensitivity of p53^−/−^ tumors to the mitochondrial inhibitor phenformin (Shackelford et al., 2013). Clearly, these results are complicated by the observation that p63 can also participate in the response to metformin (Su et al., 2012). The ability of p53 to allow cells to withstand stress and damage also extends to human cancer, where the retention of WT p53 can predict a good response in some cancers but is also associated with poor prognosis and poor response to therapy in breast tumors (Bertheau et al., 2008). Several p53 target proteins may also play dual roles. The regulation of FAO by p53 through CPT1C expression presumably helps to buffer brain cells from nutrient stress (Sanchez-Macedo et al., 2013). However, the overexpression of CPT1C (independent of p53) contributes to the survival of cancer cells under glucose deprivation or hypoxia (Zaugg et al., 2011). Similarly, the p53 target protein TIGAR is critical in the response to stress, and decreased TIGAR levels have been associated with increased migration, proliferation, and tumorigenicity in cells depleted of citrate synthase (Lin et al., 2012). On the other hand, TIGAR promotes the activity of the PPP and HK2 under hypoxia (Cheung et al., 2012), activities that would be predicted to assist tumorigenesis, and also protects against radiotherapy induced DNA damage and senescence (Peña-Rico et al., 2011). Interestingly, the activity of PFKFB4, another enzyme with phosphatase function similar to TIGAR, has recently been shown to be necessary for the survival of prostate cancer cells (Ros et al., 2012). Furthermore, the role of TIGAR in maintaining tumor cell survival is highlighted by the fact that some tumor types have elevated levels of TIGAR expression (Wanka et al., 2012; Won et al., 2012), and the inhibition of certain therapeutic targets is associated with a decrease of TIGAR expression. For example, the inhibition of c-MET, a protein that is often associated with poor patient survival, leads to a decrease in TIGAR and a subsequent increase of ROS and cell death (Lui et al., 2011).

Both p63 and p73 have been implicated in tumor suppression and protection from metastasis. Interestingly, metabolic functions that show similarities to those exhibited by p53 are now beginning to be unraveled for both these proteins. TAp63 influences glucose and lipid metabolism (Su et al., 2012), whereas TAp73 has been implicated in autophagy, the control of ROS, and the maintenance of mitochondrial complex IV (Rufini et al., 2012). However, it remains to be determined whether the metabolic functions of p63 and p73 contribute to tumor suppression (or even tumor promotion) and how they do it. The discovery that p53 family members regulate metabolism may also have important implications for tumors that express (oncogenic) mutant forms of p53, as is the case in an estimated half of all human cancers. Both p63 and p73 can be bound by these p53 mutants (Gaiddon et al., 2001), which thereby functionally deplete cells of all p53 family members. Thus, mutations in p53 not only lead to a loss of WT p53 function but also to gains of function that are associated with the inhibition of p63 and p73 function, such as the promotion of invasion and metastasis (Muller et al., 2009). Interestingly, mutant p53 also displays such a gain of function in the context of metabolism. The inhibition of autophagy by cancer-relevant p53 mutants (Morselli et al., 2008) may be mediated through the interaction of mutant p53 with p73 (Zawacka-Pankau et al., 2010). However, mutant p53 can also control metabolism independently of exerting control over p63 or p73. In the context of lipid metabolism, mutant p53 has been found to activate genes from the mevalonate pathway in breast cancer cells (Freed-Pastor et al., 2012). As mentioned above, this is achieved by binding to the transcriptional activator of cholesterol biosynthesis genes SREBP2, which does not interact with WT p53. These studies suggest that mutant p53 proteins may exert still unknown effects on cellular metabolism, either independently or via the inactivation of p53 family members, which may promote tumor development.

#### Metabolic Syndrome

Metabolic syndrome is a major health problem in industrialized societies. Characterized by obesity, insulin resistance, glucose intolerance, and diabetes, metabolic syndrome is associated with an increased risk of cardiovascular disease and cancer. Furthermore, the connection between diabetes and cancer is highlighted by several studies that show that the antidiabetic drug metformin can potentially have anticancer effects. In diabetics, metformin use is associated with a reduced risk of cancer and lower cancer mortality as well as with increased complete response rates to neoadjuvant chemotherapy in breast cancer (reviewed in Jalving et al., 2010).

Given that p53 plays an important role in the regulation of lipid metabolism, it may not be surprising that p53 has been demonstrated to be involved in systemic conditions related to lipid metabolism, such type 2 diabetes and obesity (Yahagi et al., 2003; Minamino et al., 2009; Wang et al., 2013). However, the role of p53 in metabolic disease is complex. As a form of metabolic stress, the effect of nutrient excess on the p53 pathway has received less attention than nutrient depletion. Given the presence of excess glucose and fat in the so-called “western diet” and the propensity of diabetes to induce high levels of circulating glucose, this topic warrants attention. p53 has been shown to contribute to the pathogenesis of metabolic disease in mouse models, especially under conditions of nutrient excess. High lipid levels (i.e., obesity) increase oxidative stress levels and lead to p53 induction. This activation of p53 may help to curtail lipid accumulation by enhancing lipid catabolism (Goldstein and Rotter, 2012) but could also result in insulin resistance and diabetes (Minamino et al., 2009). Indeed, adipocytes from obese mice display elevated levels of p53 (Yahagi et al., 2003), and liver p53 levels are induced in mouse models of hepatic steatosis (fatty liver disease) associated with obesity (Yahagi et al., 2004) and chronic alcohol consumption (Derdak et al., 2011). In these models, knockdown or chemical inhibition of p53 ameliorates disease. For example, p53 deficiency improves insulin sensitivity in genetically obese mice (Minamino et al., 2009). In models of steatosis, the inhibition of p53 diminished triglyceride accumulation and promoted FAO in the liver (Derdak et al., 2013). Similarly, high glucose levels inhibit AMPK activity and increase ROS generation. This leads to the upregulation of Nox4 and the activation of p53-induced apoptosis in glomerular epithelial cells (podocytes), the loss of which may contribute to albuminuria and diabetic kidney disease. The reactivation of AMPK by AICAR in this context leads to a reduction in Nox4 levels, resulting in a suppression of p53 (Eid et al., 2010). Likewise, metformin-induced AMPK and Sirt1 activation has been shown to lower p53 protein levels in hepatoma cells exposed to high glucose, whereas the overexpression of p53 in this context attenuated the effects of metformin on AMPK activation (Nelson et al., 2012). These studies illustrate the complexity and context dependence of the p53 response, given that, under condition of low glucose, AMPK activation induces p53.

However, other studies suggest that p53 can protect from the development of obesity, diabetes, and liver steatosis. In male mice on a high-fat diet, the loss of p53 resulted in a substantial increase in liver lipid accumulation and body mass in comparison to WT animals (Wang et al., 2013). Moreover, the inability to properly activate p53 has been shown to contribute to glucose intolerance. Mice carrying a p53 mutant that cannot be activated by ATM through phosphorylation develop insulin resistance and glucose intolerance, which are associated with a decreased antioxidant function (Armata et al., 2010). Mice that ectopically express Δ40p53, a p53 mutant that lacks part of the transactivation domain, also exhibit glucose intolerance, hypoinsulinemia, and defects in β cell mass and proliferation, suggesting that p53 plays a role in the regulation of β cell proliferation (Hinault et al., 2011). Conversely, super-p53 mice, which express an additional copy of normally regulated WT p53, have been found to display improved glucose tolerance (Franck et al., 2013). Another way in which p53 may be involved in the protection from diabetes is by protecting preadipocytes from ROS. Adipocytes are important in the maintenance of metabolic homeostasis and protection from lipotoxicity. p53 has been suggested to help adipocytes handle the effects of lipid and cholesterol overload, thereby maintaining adipocyte viability (Bazuine et al., 2009).

These contradictory results suggest that the outcome of p53 activation is tissue specific and most likely depends on the type of stress. Another possibility is that basal levels of p53 help in maintaining lipid homeostasis (Goldstein and Rotter, 2012) and protect from metabolic disease, whereas the deregulation of p53 or chronic p53 activation by sustained metabolic stress (such as nutrient excess or obesity) may ultimately contribute to disease pathogenesis. Recently, depletion of TAp63 has been shown to result in obesity and symptoms of type 2 diabetes in both aging mice and mice consuming a high-fat diet. The protective effects of TAp63 are thought to be mediated via the transcriptional activation of *Sirt1*, *AMPKα2*, and *LKB1* (Su et al., 2012). On the other hand, knockdown of TAp73 resulted in an increase in insulin sensitivity and protection from glucose intolerance in mice on a high-fat diet, possibly because of the positive effects of ROS on insulin signaling (Rufini et al., 2012). Once again, these observations underscore the complex role of the p53 family during the development of metabolic syndrome.

#### Aging

Aging is characterized by functional decline and is closely linked to the development of cancer, neurodegeneration, and metabolic disease. p53 is thought to play an important but complex role in the regulation of aging and longevity, and contradictory studies have shown that p53 expression can both promote and inhibit premature aging. Initial studies showed that mice carrying one truncated p53 mutant allele that exhibits increased p53 activity (p53^+/m^ mice) had a shortened lifespan (Tyner et al., 2002). Similarly, mice expressing a naturally occurring 44 kD truncated isoform of p53 displayed a reduced longevity and symptoms of early aging (Maier et al., 2004). However, more recent studies suggest that these early aging phenotypes are caused by abnormal and chronic enhancement of p53 activity, whereas normally regulated p53 could increase longevity (Hinkal et al., 2009; Matheu et al., 2007). Indeed, neither super-p53 mice nor mice in which p53 is stabilized as a result of low MDM2 activity displayed early-aging phenotypes (García-Cao et al., 2002; Mendrysa et al., 2006). Moreover, super-p53 mice that also carried an additional p19^ARF^ allele display a hyperresponsive p53 pathway, which results in increased longevity (Matheu et al., 2007). The underlying mechanisms by which p53 regulates aging are not completely understood. The ability of p53 to combat oxidative stress by inducing antioxidant responses and maintaining mitochondrial integrity could promote longevity and protect from early aging (Maddocks and Vousden, 2011). Similarly, the ability of p53 to negatively regulate the mTOR and AKT signaling pathways could contribute to a longevity phenotype, given that this would mimic the effects of caloric restriction (Gudkov et al., 2011). In support of this hypothesis, the inhibition of mTOR by p53 has been shown to switch the ultimate outcome of the p53 response from irreversible senescence to reversible quiescence, whereas the inhibition of mTOR by rapamycin mimicked the suppression of senescence by p53 (Korotchkina et al., 2010). Therefore, it has been hypothesized that p53 regulates senescence (and thereby aging) via a two-step mechanism (Gudkov et al., 2011) in which the activation of p53 by genotoxic or oncongenic stress leads to a cell-cycle arrest, whereas the decision on the irreversibility of this arrest depends on the inhibition of mTOR. Conversely, it has been proposed that, upon DNA damage (for example, by telomere dysfunction), p53 promotes aging through the repression of PGC-1α and PGC-1β, resulting in mitochondrial dysfunction. This sustains a feedforward cycle by increasing DNA damage (for example, through increased ROS generation), followed by sustained p53 activation, additional mitochondrial compromise, and so on, ultimately resulting in increased apoptosis, senescence, and aging (Sahin and DePinho, 2012).

Both p53 family members have also been implicated in the regulation of aging and longevity, albeit through different mechanisms. Selective depletion of TAp73 results in premature aging associated with mitochondrial dysfunction (due to decreased complex IV activity) and increased levels of ROS (Rufini et al., 2012). Furthermore, both the selective knockout of TAp63 (Su et al., 2009) and the complete deletion of p63 (Keyes et al., 2005) result in accelerated aging, suggesting a role for TAp63 in the regulation of aging. Although TAp63^−/−^ mice show some of the same premature aging defects as TAp73^−/−^ mice, the aging phenotype in TAp63^−/−^ mice is thought to be caused by a hyperproliferation of stem cells (Su et al., 2009). Another intriguing possibility is that the inhibition of p63 and p73 by mutant p53 plays a role in the proaging phenotypes of p53^+/m^ mice (Flores and Lozano, 2012). However, additional work is needed in order to elucidate the mechanisms by which the p53 family regulates aging and longevity.

### Future Directions

The metabolic functions of p53 are emerging as critical not only for tumor suppression but also for maintaining normal cellular homeostasis. Nevertheless, many questions remain. Clearly, different stress signals can activate different p53 responses, as measured by transcriptional profiles, and several proteins that contribute to the ability of p53 to promote adaptation to metabolic stress are preferentially activated by p53 in response to nutrient deprivation (Sen et al., 2011). However, little is known about the molecular mechanisms that govern the outcome of the p53 response. Although the induction of p53 after genotoxic damage generally depends on the stabilization of p53, this does not seem to be a universal requirement for the activation of a metabolic p53 response. During serine starvation, for example, recruitment of p53 to the p21 promoter and robust induction of p21 expression are observed in response to modest stabilization of p53 (Maddocks et al., 2013). Most likely, posttranslational modifications of p53 during different types and intensities of stress contribute to p53 target gene selection (Vousden and Prives, 2009; Smeenk and Lohrum, 2010). However, as discussed above, to reach various end points (or cell-fate decisions), much may depend on the interaction of p53 with critical transcription factors in the regulation of metabolism. Future studies into the mechanisms that activate p53 and mediate the p53 response under conditions of metabolic stress will not only increase our understanding of the regulation of metabolism by p53 but may also shine light on the complex role of the metabolic functions of p53 in pathologies such as cancer and diabetes. Finally, delineating how metabolism may be regulated by the p53 family members p63 and p73 will also be important for understanding the possible metabolic roles of oncogenic mutant forms of p53. The extent to which p53 family members interact with lipid and carbohydrate metabolism, cell growth, and the process of autophagy is complicated and context dependent. Additional insight into these relationships will increase our understanding of how these interactions influence health and disease and, potentially, how they can be manipulated for our benefit.
